# Reasons why self-referring patients attend the emergency department during daytime differ among socioeconomic groups: A survey from Flanders

**DOI:** 10.1080/13814788.2018.1521388

**Published:** 2018-10-30

**Authors:** Jens Detollenaere, Julie Boucherie, Sara Willems

**Affiliations:** Department of Family Medicine and Primary Health Care, Ghent University, Ghent, Belgium

**Keywords:** Emergency department, reasons, socioeconomic, referral, GP

## Abstract

**Background:** Numerous studies have shown that during out-of-hours vulnerable patients (regarding low-education and unemployment) are more likely to seek medical help in the emergency department (ED). However, little is known about why patients seek help in the ED during daytime hours and if these reasons differ among self-referring socioeconomic groups.

**Objectives:** To identify the reasons why patients opt for the ED during daytime hours when primary care services are available and identify possible social differences between socioeconomic groups.

**Methods:** In 2014–2015, trained fieldworkers surveyed 723 patients visiting four EDs in Flanders using a structured interview. These quantitative data were analysed using descriptive and logistic regression analyses.

**Results:** More than one-third of the self-referring patients reported that they attend the ED during daytime hours because they perceive their (health) problem as urgent and expect they need advanced diagnostic testing. Self-referred and low-educated patients have a 1.8 higher chance (compared to their higher-educated counterparts) of attending the ED because they expect advanced diagnostic testing. Self-referred and unemployed patients have a 3.6, 2.5 and 4.4 higher chance (compared to their employed counterparts) to opt for the ED because it is their usual source of care, family/friends refer them or they postpone care too long, respectively.

**Conclusion:** We found sociodemographic differences in motives why self-referring patients in Flanders opt for the ED during daytime hours. In general, self-referring patients attend the ED because they perceive their condition as urgent and think they may need advanced diagnostic testing.

KEY MESSAGES

- In Flanders, self-referring patients attend the ED during the daytime because they perceive their condition as urgent and think they may need advanced diagnostic testing.
- Compared to other patients, low-educated and unemployed patients more often mention care postponed too long, ED as usual care source, and referral by friends or family as motives to visit the ED during the daytime.

## Introduction

The emergency department (ED) has become an increasingly common used source of care. From 2009–2012, the number of ED visits in Belgium increased by 6.7% (290 ED visits per 1000 population in 2012) [1]. The incidence in Belgium is higher than in neighbouring countries (124, 279, and 264 ED visits per 1000 population in the Netherlands, France, and England, respectively) [1].

The Belgian healthcare system is organized according to the principles of a compulsory social health insurance system [2]. Patients are free to choose their healthcare professional and the place of treatment with this mandatory health insurance [3]. For instance, when patients suffer from an illness, they have access to any general practitioner (GP), specialist or even the ED at any time and with any (health) problem. For regular primary care consultation, this reimbursement amounts to €9.23 (co-payment of €6 for the patient). Additionally, the Belgian government and insurance companies provide some social safety nets for vulnerable populations, e.g. the regularization of the third party payment in primary care. In the third-party payment, the sickness fund pays the provider directly and the patient is only responsible for paying any co-payments, supplements or non-reimbursed services [3]. The co-payment for self-referring patients at the ED is €20.21, or €4.5 with referral. Patients who attend primary care pay the costs upfront, while patients who attend the ED receive the invoice afterwards. Despite efforts to make Belgian primary care (financially) accessible and redirecting patients from the ED to primary care, healthcare professionals do not refer 71% of the patients to the ED. Many of these patients could be treated in primary care [1]. Furthermore, in the literature ED usage is positively associated with low socio-economic status [4]. People with lower education, lower income or foreign nationality are more likely to visit the ED [4–10]. Identifying the reasons why self-referring patients attend the ED is essential for quality and population health improvement but also regarding increased cost and workload control [11,12]. Most common reasons for self-referrals to the ED are that patients believe their problem requires immediate care; the primary care system is not accessible; and the patients have more trust in the ED than in the primary care services are the most commonly reported [12–22]. However, since most of these studies were conducted out-of-hours, it is not clear whether these reasons are the same during daytime hours (i.e. primary care facilities are supposed to be more easily accessible).

This study aims to explore why patients consult the ED without referral during daytime hours when primary care services are available and to identify social differences in the reasons why patients consult. To the best of our knowledge, we are the first to conduct this study exclusively on consultation during daytime hours. As the French and German healthcare systems are similar to the Belgian system, results of current study could be relevant for these countries. In these mainly Bismarckian systems, patients are not obliged to register with a regular GP and the GP does not control access to secondary care (but have financial incentives to register with a GP and obtain a referral) [23].

## Methods

### Study design

We conducted a cross-sectional multicentre study to study the reasons why self-referring patients attend the ED during daytime hours. Trained interviewers collected between September 2014 and March 2015, during daytime hours (Monday-Friday, between 8.00 a.m. and 6.00 p.m.) at four EDs in Flanders, Belgium.

### Ethics

Ethical approval for the study was acquired by the Ethics Committee of Ghent University Hospital, and additionally approved by the Ethics Committee of the Zeno general hospital, Sint-Lucas general hospital and the Groeninge general hospital. The Sint-Andries hospital accepted the approval by the Ethics Committee of Ghent University Hospital.

### Setting

Between September 2014 and March 2015, data were gathered at the ED of the Zeno general hospital (Knokke-Heist). From July to September 2015, data were collected at the ED of the Sint-Andries hospital (Tielt), the ED of the Sint-Lucas general hospital (Ghent) and the ED of the Groeninge general hospital (Kortrijk).

### Recruitment of patients

Trained fieldworkers were instructed to invite all adult patients (≥18 years) presenting at the EDs mentioned above to participate in the study. Participants should not have been referred by a GP, nor been suffering from a life-threatening or urgent health condition and should not have entered the ED by ambulance or mobile urgency group (MUG). The MUG is a mobile medical team (consisting out of a specialized doctor and specialized nurse) that provides urgent medical assistance in the event of an emergency when a patient needs medical supervision. Consecutive patients were excluded when they attended the ED for the second time during the inclusion period.

### Data collection

Data was collected by face-to-face survey interview. The questionnaire included sociodemographic information and a list of 16 reasons that were based on the dimensions of the behavioural model of access to healthcare [24,25] and reasons reported in other studies [4–22,26]. See Table 1 for reasons and their abbreviations throughout this article. Face validity of the questions was tested using cognitive interviewing with ten respondents. Questionnaires were in Dutch and translated into French, English, Turkish, and Arabic using a forward-backward translation procedure.

**Table 1.** Reasons to opt for the emergency department (ED) and their abbreviations in this article and percentage of patients who ticked this reason.

Reason	Abbreviation	% (*n* = 723)
I usually visit the ED with my (health) problems.	Usual source of care	5.6
I have delayed care too long, so my problem can only be solved by care of the ED.	Postponed seeking care too long	6.5
I do not have to pay during my visit to the ED.	Financial motives	7.6
The ED is the most easily accessible for me (e.g. regular buses or trams).	Accessibility	10%
I do not have to wait long here.	Waiting time	14.7
My family/friends advised me to go to the ED.	Family/friends	16.9
Other	Other	17.8
Given my medical history, the ED is the most appropriate choice for my problem.	Medical history	18.1
I first called my GP, but I could not reach her/him.	Could not reach GP	18.5
The ED provides the best care.	Best care	19.4
The ED was the closest healthcare facility for me.	Proximity	21.3
I did not know where else to go with this problem.	Did not know where else to go	22.1
I have already visited the ED in the past.	Experience	27.4
I am satisfied with the care that is provided at the ED.	Satisfaction	28.8
Given the seriousness of my problem, I think that the ED can give me the best and most appropriate care.	Seriousness of the problem	33.2
I think that additional (medical) and advanced test will be necessary.	Advanced diagnostic tests	36.9

The English questionnaire is available as supplemental material online.

### Variables

Respondents were asked to tick the most relevant reason for consulting the ED that day. To determine if the reasons for attending the ED without GP referral differed between socio-economic groups, the following three variables were entered in the regression models: educational level, employment, and having financial problems. Highest educational attainment was recoded into three categories: low (no diploma, primary school, and the first half of secondary school), middle (secondary school) and high education (higher education). Middle-educated patients were entered into the model as the reference category. Employment was recoded into three categories: no paid job (paid suspended employment and unemployment), retirement, student, and paid job (reference category). Having financial problems was dichotomized: no financial difficulties experienced by the respondent (very easy or easy to make ends meet at the end of the month) and financial difficulties (difficult or very difficult to make ends meet). No financial difficulties were entered as the reference category. Furthermore, we controlled for sex, age, and having a regular GP in the statistical models. For sex, male patients (reference category) were compared to female patients. Because the variable age was rejected by the normal distribution hypothesis (using the Shapiro–Wilk test), this variable was logarithmically transformed. Concerning the regular GP, patients were asked if they had a regular GP (yes/no). Having no regular GP was the reference category. Multicollinearity between the independent variables was tested by calculating the variance inflation factors (VIF). No multicollinearity was found.

### Data analysis

Using multiple logistic regression modelling, the relative contribution of all independent variables on reasons for attending the ED was assessed. The analyses were controlled for the location of the ED by adding a dummy for every ED to the statistical models. Due to multiple testing in the statistical models, the level of significance was lowered from the conventional *P* ≤ .05 to *P* ≤ .01 (99%CI reported). This adjustment does not eliminate multiple testing problems; however, it lowers the chance of it occurring.

## Results

In this study, 723 patients participated in which 55.5% were men. Mean age of the respondents was 47 years. Virtually all participants had a regular GP. Table 2 shows the details of the descriptive statistics. All VIF values were below three indicating that the independent variables did not interfere with each other.

**Table 2.** Descriptive statistics.

	*n* (%)
Gender (*n* = 721)	
Male	400 (55.3)
Age (*n* = 723)	
18–35 years	226 (31.3)
36–55 years	270 (37.3)
>56 years	227 (31.4)
Educational level (*n* = 703)	
Low	245 (33.9)
Middle	313 (43.3)
High	145 (20.1)
Employment (*n* = 699)	
Paid job	438 (60.6)
No paid job	70 (9.7)
Retirement	151 (20.9)
Regular GP (*n* = 710)	
Yes	669 (92.5)
Financial problems (*n* = 684)	
Financial problems	171 (23.7)

Table 1 presents an overview of the different reasons why patients opt for the ED during daytime hours without the referral of a GP. The most frequent indicated reasons are the expectation that advanced diagnostic tests will be needed (36.9%), perceived seriousness of the problem (33.2%) and prior satisfaction with the offered ED care (28.8%). In contrast, the least indicated reasons for attending the ED are financial motives (7.6%), care that has been delayed for too long (6.5%) and the ED being the usual source of care (5.6%).

Below, we will only report the significant results of the logistic regression models showing the association between the reasons for opting for the ED (dependent variable) and the socio-economic determinants (independent variables). See Supplemental material, available online, for non-significant results.

The analyses reveal that the odds for choosing the ED because of the expectation that advanced diagnostic tests are required is higher for low-educated patients compared to their middle-educated counterparts (OR: 1.82; 99%CI: 1.07–3.10).

Unemployment is significantly associated with several reasons for choosing the ED without referral. Patients who are not employed were more likely to indicate that they attend the ED because the ED is their usual source of care (OR: 3.64; 99%CI: 1.04–12.79), family or friends referred them to the ED (OR: 2.52; 99%CI: 1.11–5.71) or they postponed seeking care too long (OR: 4.40; 99%CI: 1.43–13.55).

## Discussion

### Main findings

This study shows that self-referring patients most frequently attend the ED because they perceive their condition as urgent and will need advanced diagnostic testing. Furthermore, the present study indicates that vulnerable groups (regarding low-education and no-employment) are more likely to bypass the GP because they postponed seeking care too long, for financial motives, because the ED is their usual source of care or for their medical history.

### Reasons for attending the ED without referral

Our study found that most of the participants opt for the ED because they expected to need advanced diagnostic testing. This result is in line with previous literature, showing that patients are convinced they need advanced radiologic and/or laboratory investigations to get a diagnosis [12–14,22]. Given that all these advanced diagnostic tests can be done in one place, it is somewhat logical to by-pass the GP and go straight to the ED, potentially reducing costs by doing so [8]. The second most indicated reason why participants directly opt for the ED is the feeling that their condition is serious/urgent and cannot wait to be treated. This finding is also in agreement with previous studies, underscoring the difficulties patients perceive in determining the seriousness of their condition [12,14,19]. However, determining which (health) problems should be treated at the ED is a long-lasting debate, even among healthcare professionals [15,19,27], which highlights the potential danger of turning away inappropriate or non-emergency problems [14]. Our questionnaire did not provide data about the seriousness of the reason for the encounter, making it impossible to consider this in the analyses. In addition, one-fifth of the respondents reports not knowing where else to go with their health problem. The fact that data was collected during office hours (i.e. when other healthcare facilities are available) should function as an alarm for policymakers that patients do not find their way easily in the healthcare system, leading to a failure of the healthcare system to deliver efficient and timely care to the entire population. Altogether, these findings might also mirror a knowledge deficit among patients, e.g. incorrect evaluation when a condition requires care and which facility is the most suitable, etc. Low health literacy is associated with reduced patient safety, less prevention, increase in hospitalizations, worse health outcomes, and increased mortality risk [28]. Inaccessibility to understandable information or healthcare is seldom or never exclusively attributable to patients, healthcare professionals, or the healthcare system. It is rather a mismatch of the patient’s ability to understand health-related information and the healthcare provider or health system’s response [28,29].

### Socio-economic differences in reasons for attending the ED without referral

Despite existing social protection mechanisms (maximum billing), policymakers should also accommodate the accessibility of the primary care system for unemployed citizens, as our results suggest that unemployed citizens are more likely to attend the ED as it is their regular source of care, because they postponed seeking care too long, or family or friends refer them. This might seem like a contradiction, self-referring patients pay much more at the ED (€20.21) compared to referred patients (€10.50). Liquidity constraints might push these patients into postponing care (health problems might get worse so that the ED becomes the only appropriate choice), and into ED usage. However, since October 2015, the Belgian GPs are obliged to apply the third-party scheme for low-income citizens, hoping to make the healthcare system more accessible for vulnerable groups. Last, results show that low-educated patients are more likely to opt for the ED because they expect that advanced diagnostic testing will be necessary.

### Strengths and limitations

Merits of the present study lie in the fact that, to the best of our knowledge, this study is the first to address these two research aims during daytime hours. During the day, patients have a wide variety of other healthcare facilities that easy available (e.g. GP in the primary care system). However, to compare reasons during office hours and after-hours, future research should include data collection during the entire day (including office hours and after-hours). An important limitation to keep in mind is that data was collected during 2014–2015 and data collection ended just before the rollout of the obligatory implementation of the third-party scheme, we encourage future studies to evaluate the (longitudinal) effect on ED care. Furthermore, five fieldworkers collected data. Although they all received an extensive one-on-one introduction in data collection, confidentially and deliverables, it is possible that several factors or characteristics of the fieldworker biased the data collection. Fieldworkers were asked to remind the respondents to tick only the most important reason to choose for the ED; however, it was possible for the respondents to tick multiple reasons. Every ticked reason was analysed as a separate unit, which could have biased the results.

### Implications

Findings of the current study might mirror a knowledge deficit among patients, e.g. incorrect evaluation when a condition requires care and which care facility is the most suitable. In Belgium, 71% of the ED patients are not referred by a healthcare professional [1]. One could potentially argue that Belgian EDs are too easy to access. There is no gatekeeper to the healthcare system and no hard financial consequences when attending the ED without referral. In the Netherlands, for example, the percentage of self-referred patients is substantially lower (17.4%) [30]. Dutch policymakers direct patients to the most suitable healthcare facility by a two-folded approach: (i) the GP functions as a gatekeeper and (ii) access to the GP is free. Belgian policymakers could implement this two-fold approach to make the healthcare system more efficient and more equitable.

## Conclusion

We found sociodemographic differences in motives why self-referring patients in Flanders opt for the ED during daytime hours. In general, self-referring patients attend the ED because they perceive their condition as urgent and think they may need advanced diagnostic testing.

## Supplementary Material

Supplemental Material
