# Supplementary material for: Reasons why self-referring patients attend the emergency department during daytime differ among socioeconomic groups: A survey from Flanders
Source: Eur J Gen Pract. 2018 Oct 30;24(1):246–51. doi: 10.1080/13814788.2018.1521388 (PMC6211319; doi:10.1080/13814788.2018.1521388)
Supplement: Supplemental Material [file IGEN_A_1521388_SM1087.docx]

**APPENDIX**

**Appendix 1** Questionnaire

| **Appendix 2.** Results of logistic regression modelling, odds ratio (OR) and confidence interval (C.I) reported (analyses controlled for location of the hospital) | | | | | | | | | | | | | |
| --- | --- | --- | --- | --- | --- | --- | --- | --- | --- | --- | --- | --- | --- |
|  | **Low education versus *middle education (ref.)*** | | **High education versus *middle education (ref.)*** | | **No paid job versus *paid job (ref.)*** | | **Retirement versus *paid job (ref.)*** | | **Student versus *paid job (ref.)*** | | **Financial difficulties versus *no financial difficulties (ref.)*** | | **Included N** |
|  | **OR** | **C.I.** | **OR** | **C.I.** | **OR** | **C.I.** | **OR** | **C.I.** | **OR** | **C.I.** | **OR** | **C.I.** |  |
| Waiting ^1^ | 0.77 | [0.38-1.56] | 1.14 | [0.51-2.56] | 1.59 | [0.62-4.03] | 1.27 | [0.45-3.61] | 0.53 | [0.08-3.75] | 1.33 | [0.68-2.60] | 658/723 |
| Did not know where else ^2^ | 1.14 | [0.61-2.13] | 1.57 | [0.78-3.20] | 1.22 | [0.53-2.83] | 1.21 | [0.48-3.04] | 1.09 | [0.30-3.94] | 1.10 | [0.61-1.99] | 658/723 |
| Experience ^3^ | 0.74 | [0.44-1.37] | 0.85 | [0.43-1.69] | 0.94 | [0.42-2.14] | 1.25 | [0.53-2.92] | 1.66 | [0.49-5.62] | 1.25 | [0.72-2.19] | 658/723 |
| Satisfaction ^4^ | 0.88 | [0.50-1.54] | 0.93 | [0.48-1.82] | 0.88 | [0.40-1.98] | 1.51 | [0.65-3.51] | 0.64 | [0.15-2.74] | 1.35 | [0.78-2.33] | 658/723 |
| Usual source of care ^5^ | 0.37 | [0.12-1.16] | 0.47 | [0.12-1.94] | **3.64** | **[1.04-12.79]** | 2.22 | [0.45-11.02] | 5.19 | [0.58-46.59] | 0.48 | [0.15-1.52] | 658/723 |
| Family/friends ^6^ | 1.09 | [0.55-2.15] | 0.95 | [0.41-2.22] | **2.51** | **[1.11-5.71]** | 1.22 | [0.42-3.60] | 0.87 | [0.19-3.90] | 1.00 | [0.51-1.95] | 658/723 |
| Financial motives ^7^ | 0.74 | [0.30-1.87] | 0.55 | [0.16-1.84] | 1.71 | [0.49-5.98] | 3.51 | [0.89-13.95] | 1.78 | [0.23-13.98] | 0.83 | [0.33-2.10] | 658/723 |
| Proximity ^8^ | 1.17 | [0.63-2.19] | 1.36 | [0.65-2.83] | 2.16 | [0.96-4.83] | 1.85 | [0.74-4.64] | 1.05 | [0.24-4.61] | 1.01 | [0.55-1.87] | 658/723 |
| Best care ^9^ | 1.03 | [0.54-1.95] | 1.02 | [0.48-2.17] | 0.99 | [0.41-2.42] | 1.22 | [0.47-3.15] | 0.51 | [0.07-3.60] | 0.92 | [0.49-1.73] | 658/723 |
| Medical history ^10^ | 0.62 | [0.31-1.21] | 0.85 | [0.39-1.85] | 1.60 | [0.65-3.93] | 1.74 | [0.67-4.55] | 2.28 | [0.56-9.30] | 0.82 | [0.42-1.59] | 658/723 |
| Seriousness ^11^ | 1.01 | [0.57-1.77] | 1.25 | [0.64-2.43] | 0.81 | [0.36-1.83] | 0.72 | [0.31-1.66] | 1.60 | [0.46-5.60] | 0.94 | [0.54-1.64] | 658/723 |
| Postponed care seeking too long ^12^ | 0.71 | [0.26-1.96] | 1.51 | [0.51-4.42] | **4.40** | **[1.43-13.55]** | 3.01 | [0.71-12.77] | 0.84 | [0.05-13.24] | 0.87 | [0.33-2.28] | 658/723 |
| Advanced diagnostic tests ^13^ | **1.82** | **[1.07-3.10]** | 1.45 | [0.77-2.74] | 0.77 | [0.35-1.68] | 1.41 | [0.64-3.11] | 1.89 | [0.66-5.42] | 1.15 | [0.69-1.93] | 658/723 |
| Transport ^14^ | 1.34 | [0.59-3.04] | 0.81 | [0.27-2.43] | 2.55 | [0.94-6.92] | 1.30 | [0.39-4.33] | 0.85 | [0.06-12.99] | 1.40 | [0.65-2.98] | 658/723 |
| Could not reach GP ^15^ | 1.55 | [0.75-3.22] | 1.63 | [0.70-3.81] | 0.80 | [0.27-2.41] | 1.50 | [0.54-4.16] | 0.95 | [0.13-6.91] | 1.52 | [0.77-2.97] | 658/723 |
| Other ^16^ | 1.19 | [0.62-2.30] | 1.23 | [0.56-2.73] | 1.11 | [0.47-2.60] | 0.50 | [0.17-1.48] | 1.22 | [0.30-4.93] | 1.33 | [0.71-2.50] | 658/723 |

Abbreviations in this article: ^1^ I do not have to wait long here, ^2^ I did not know where else to go with this problem, ^3^ I have already visited the ED in the past, ^4^ I am satisfied with the care that is provided at the ED, ^5^ I usually visit the ED with my (health) problems, ^6^ My family/friends advised me to go to the ED, ^7^ I do not have to pay during my visit to the ED, ^8^ The ED was the closest healthcare facility for me, ^9^ The ED provides the best care, ^10^ Given my medical history, the ED is the most appropriate choice for my problem, ^11^ Given my medical history, the ED is the most appropriate choice for my problem, ^12^ I have delayed care too long, so my problem can only be solved by care of the ED, ^13^ I think that additional (medical) and advanced test will be necessary, ^14^ The ED is the most easily accessible for me (e.g. regular buses or trams), ^15^ I first called my GP, but I could not reach her/him, ^16^ Other reason.

| **Appendix 2.** Results of logistic regression modelling, odds ratio (OR) and confidence interval (C.I) reported (analyses controlled for location of the hospital) – *continued* | | | | | | |
| --- | --- | --- | --- | --- | --- | --- |
|  | **Women versus *men (ref.)*** | | **Age** | | **Regular GP versus *no regular GP (ref.)*** | |
|  | **OR** | **C.I.** | **OR** | **C.I.** | **OR** | **C.I.** |
| Waiting ^1^ | 0.67 | [0.37-1.23] | 1.00 | [0.98-1.03] | 1.68 | [0.45-7.74] |
| Did not know where else ^2^ | 1.12 | [0.67-1.86] | 1.00 | [0.98-1.02] | 0.44 | [0.18-1.09] |
| Experience ^3^ | 0.76 | [0.47-1.25] | 1.01 | [0.99-1.03] | 0.75 | [0.28-2.00] |
| Satisfaction ^4^ | 0.71 | [0.44-0.15] | 1.01 | [0.99-1.03] | 1.14 | [0.41-3.14] |
| Usual source of care ^5^ | 0.87 | [0.34-2.24] | 1.00 | [0.97-1.04] | 0.69 | [0.12-4.05] |
| Family/friends ^6^ | 1.41 | [0.79-2.50] | 0.99 | [0.97-1.02] | 0.88 | [0.28-2.81] |
| Financial motives ^7^ | 0.70 | [0.31-1.59] | 0.99 | [0.96-1.03] | 1.44 | [0.19-10.7] |
| Proximity ^8^ | 0.75 | [0.45-1.28] | 1.00 | [0.98-1.02] | 2.08 | [0.57-7.60] |
| Best care ^9^ | 0.75 | [0.44-1.30] | 1.10 | [0.99-1.03] | 0.90 | [0.29-2.71] |
| Medical history ^10^ | 0.79 | [0.45-1.39] | 1.02 | [0.99-1.04] | 0.53 | [0.18-1.53] |
| Seriousness ^11^ | 0.78 | [0.48-1.24] | **1.03** | **[1.01-1.05]** | 2.45 | [0.77-7.87] |
| Postponed seeking care too long ^12^ | 0.87 | [0.38-2.00] | 0.99 | [0.96-1.03] | 1.78 | [0.24-13.11] |
| Advanced diagnostic tests ^13^ | 0.96 | [0.62-1.49] | 1.01 | [0.99-1.03] | 1.84 | [0.68-5.01] |
| Transport ^14^ | 1.00 | [0.49-2.02] | 1.02 | [1.00-1.05] | 1.43 | [0.26-7.86] |
| Could not reach GP ^15^ | 0.64 | [0.35-1.17] | 1.02 | [1.00-1.05] | 2.61 | [0.51-13.51] |
| Other ^16^ | 1.53 | [0.88-2.67] | 1.00 | [0.98-1.03] | 0.58 | [0.21-1.61] |
